# Management of Osteoporosis in Men: A Narrative Review

**DOI:** 10.3390/ijms222413640

**Published:** 2021-12-20

**Authors:** Fabio Vescini, Iacopo Chiodini, Alberto Falchetti, Andrea Palermo, Antonio Stefano Salcuni, Stefania Bonadonna, Vincenzo De Geronimo, Roberto Cesareo, Luca Giovanelli, Martina Brigo, Francesco Bertoldo, Alfredo Scillitani, Luigi Gennari

**Affiliations:** 1 Endocrinology and Metabolism Unit, University-Hospital S. Maria della Misericordia, 33100 Udine, Italy; fabio.vescini@asuiud.sanita.fvg.it (F.V.); antoniostefano.salcuni@asufc.sanita.fvg.it (A.S.S.); 2 Istituto Auxologico Italiano, IRCCS, 20149 Milan, Italy; a.falchetti@auxologico.it (A.F.); s.bonadonna@auxologico.it (S.B.); 3 Department of Medical Biotechnology and Translational Medicine, University of Milan, 20122 Milan, Italy; luca.giovanelli@unimi.it; 4 Unit of Endocrinology and Diabetes, Campus Bio-Medico University, 00128 Rome, Italy; a.palermo@unicampus.it; 5 Unit of Endocrinology—Policlinico Morgagni CCD, 95125 Catania, Italy; vdg@iol.it; 6 Center of Metabolic Disease, S.M. Goretti Hospital, 04100 Latina, Italy; robertocesareo@libero.it; 7 Department of Medicine, University of Verona, 37129 Verona, Italy; martina.brigo@gmail.com (M.B.); francesco.bertoldo@univr.it (F.B.); 8 Unit of Endocrinology, Ospedale “Casa Sollievo della Sofferenza”, IRCCS, San Giovanni Rotondo, 71013 Foggia, Italy; alfredo.scillitani@gmail.com; 9 Department of Medicine, Surgery and Neurosciences, University of Siena, 53100 Siena, Italy; luigi.gennari@unisi.it

**Keywords:** osteoporosis, BMD, male, bone fragility, fractures, DXA

## Abstract

Male osteoporosis is a still largely underdiagnosed pathological condition. As a consequence, bone fragility in men remains undertreated mainly due to the low screening frequency and to controversies in the bone mineral density (BMD) testing standards. Up to the 40% of overall osteoporotic fractures affect men, in spite of the fact that women have a significant higher prevalence of osteoporosis. In addition, in males, hip fractures are associated with increased morbidity and mortality as compared to women. Importantly, male fractures occur about 10 years later in life than women, and, therefore, due to the advanced age, men may have more comorbidities and, consequently, their mortality is about twice the rate in women. Gender differences, which begin during puberty, lead to wider bones in males as compared with females. In men, follicle-stimulating hormones, testosterone, estrogens, and sex hormone-binding levels, together with genetic factors, interact in determining the peak of bone mass, BMD maintenance, and lifetime decrease. As compared with women, men are more frequently affected by secondary osteoporosis. Therefore, in all osteoporotic men, a complete clinical history should be collected and a careful physical examination should be done, in order to find clues of a possible underlying diseases and, ultimately, to guide laboratory testing. Currently, the pharmacological therapy of male osteoporosis includes aminobisphosphonates, denosumab, and teriparatide. Hypogonadal patients may be treated with testosterone replacement therapy. Given that the fractures related to mortality are higher in men than in women, treating male subjects with osteoporosis is of the utmost importance in clinical practice, as it may impact on mortality even more than in women.

## 1. Introduction

The public health burden due to osteoporosis and fragility fractures is progressively rising as the ageing population increases in many countries. Although a 50-year-old Caucasian woman has a lifetime risk of fracture of 40% [1], the prevention and management of the disease are still insufficient and, as a matter of fact, many studies have revealed that people at high fracture risk do not receive adequate treatment [2]. As osteoporosis is commonly considered to be a female disease, it is clear that, in men, whose lifetime risk of fracture at the age of 50 is around the 13% [1], it is even more underestimated [3]. A small proportion of men with fractures are correctly screened for osteoporosis and consequently treated with the appropriate drugs [3]. 

The present paper is not intended to be a systematical review of the literature on male osteoporosis. As far as an enormous number of papers has been produced on this matter, we aimed to create an easy and practical narrative review for the colleagues looking for a summary on male osteoporosis, particularly for those approaching it for the first time.

## 2. Pathophysiology

The gender differences between male and females begin during puberty, when in girls estrogens (E) inhibit periosteal and promote endocortical bone formation, thus limiting bone diameter, while, in boys, testosterone (T) leads to an increase in bone diameter by increasing periosteal apposition. During aging, men have a greater periosteal apposition and similar endocortical resorption than women and, thus, a lower net bone loss [4].

Androgens act on bone both directly through the androgen receptor (AR) and indirectly by activating E receptor-α or -β (ERα or ERβ) thanks to their aromatization into E. The periosteal bone expansion requires both the AR and ERα pathways, while the AR signaling is the main mechanism for normal trabecular bone development in males. These considerations explain the results of several studies, indicating the important role of E in the regulation of bone loss and bone metabolism in men, and particularly the ability of aromatizing T into E [5,6]. Indeed, in men, low serum E and T and high serum sex-hormone binding globulin (SHBG) predict clinical vertebral, nonvertebral, and hip osteoporosis fractures [7].

In addition to the well-known impact of reduced T, E, and SHBG levels, they play a significant role in skeletal remodeling and strength, together with a follicle-stimulating hormone (FSH). In fact, FSH can directly stimulate bone resorption [8,9,10,11,12] by binding to its specific receptor on osteoclasts. In particular, FSH seems both to upregulate the expression of RANK, namely the receptor of RANK-L, which is a key cytokine in osteoclastic differentiation, survival, and activity [13,14], and to indirectly stimulate bone resorption by releasing osteoclastogenic cytokines [15]. Furthermore, FSH may also negatively regulate osteoblast differentiation from mesenchymal stem cells [16]. In fact, a polyclonal antibody generated against FSHβ has been proven to block osteoclastogenesis in vitro and, when injected in ovariectomized mice, to attenuate bone loss; not only by inhibiting bone resorption, but also by stimulating accrual [11,17].

These data are in keeping with the evidence that elevated FSH values in females correlate both with increased serum markers of bone turnover and with reduced bone mineral density (BMD) [17,18,19,20,21]. Moreover, in males, FSH is likely to exert a direct action on the skeleton in the context of the pituitary–bone axis, as suggested by the finding of a negative correlation between FSH levels and lumbar/femoral BMD [22]. Furthermore, in patients with Klinefelter syndrome, T levels and the AR gene CAG polymorphisms, consisting of a polyglutamine stretch of variable lengths within the N-terminal domain of the receptor, encoded by a variable number of CAG triplets in exon 1 of the *AR* gene located on the X chromosome, which reduce the AR sensitivity, are not associated with osteoporosis, despite the well-known relationship between hypogonadism and reduced BMD [23].

Overall, in men, T, E, SHBG, and FSH levels interact in determining the bone mass accrual, BMD maintenance, and lifetime decrease.

## 3. Genetics Aspects

It is well known that genetic factors play a relevant role in primary osteoporosis in both genders [24,25]. Most of the available genetic data have been obtained mainly in cohorts of postmenopausal women, with only a minority of studies having been conducted on male osteoporotic subjects. However, it has been reported that the male offspring of female subjects with osteoporosis show reduced bone mass well before age-related bone loss ensues [26], suggesting the expression of inherited determinants of osteoporotic risk from an early age. Consistent with this hypothesis, studies on male twin pairs revealed an age-related increase in intra-pair differences in radial bone mass and width both in monozygotic and dizygotic pairs [27]. As in other human multifactorial pathologies, the study of “nature’s experiments”, represented by rare bone diseases (e.g., aromatase deficiency, osteoporosis pseudoglioma syndrome, etc.), has represented a useful resource in investigating the genetic pathways related to male osteoporosis. Then, less severe functional defects (namely, polymorphic variants) in the same genes or in other genes have been associated with the predisposition of osteoporosis in men [25]. 

### 3.1. Genes Encoding for Hormones with a Well-Established Role in Bone Biology

By analyzing extreme phenotypes in male osteoporotic subjects, which is useful for studying rare quantitative and qualitative traits, such as BMD and fractures, the role of estrogen clearly emerged in the pathophysiology of male osteoporosis. Specifically, Smith et al. first described a case of osteoporosis and tall stature in a young man associated to estrogenic resistance caused by a mutation in the *ER* gene [28]. This phenotype occurred despite the presence of normal or even higher-than-normal androgen levels. A similar clinical picture was lately reported in men within a context of aromatase (an enzyme complex catalyzing the conversion of androgens to E) deficiency syndrome, suggesting that inactivating mutations in the aromatase (*CYP19A1*) gene, and thus the reduced conversion of androgen precursors into E, may be relevant in the pathophysiology of male osteoporosis [6]. Following these reports, polymorphic variants of the *CYP19A1* gene have been also related to the genetic susceptibility of male osteoporosis [6,29]. 

Part of the structural skeletal abnormalities found in men with primary osteoporosis have also been related to endocrine alterations other than sex hormone action and particularly to impaired insulin-like growth factor 1 (IGF-1) in men with idiopathic osteoporosis [30]. Indeed, together with sex steroid hormones, IGF-1 has a relevant effect on long bone growth and periosteal apposition. Accordingly, not only variation in E action [26], but also higher IGF-1 levels, have been positively associated with BMD in adult men, and a particular allelic configuration of the IGF-1 gene, leading to reduced IGF-1 levels, was described in a subset of men with idiopathic osteoporosis [30,31].

### 3.2. X-Chromosome- and Maternally Inherited-Related Genes

Mutations in plastin 3 (PLS3), a protein involved in actin bundle formation in the cytoskeleton, have been recently associated with an X-chromosomal osteoporosis form. As expected, males had an early onset of osteoporotic fractures (including childhood in most cases) and were, in general, more severely affected than females.

In addition to this, mitochondrial DNA (mtDNA) abnormalities, mostly passing from the mother to the offspring, have been reported in cases of severe male osteoporosis, with mtDNA deletion spanning part of the gene coding for complex I of the respiratory mitochondria chain, probably associated to an inefficient oxidative phosphorylation, and suggesting this to have a detrimental effect on bone metabolism [32,33].

### 3.3. Other Autosomal Genes

At present, most of the genetic studies on osteoporosis have been conducted in populations including both sexes, with a clear female predominance, analyzing mainly autosomal genes involved in bone gender-unrelated molecular pathways. Genome-wide association studies (GWASs) in unselected population-based individuals, including males, have revealed several genes associated with low bone mass and risk of fracture, other than the known mutations in collagen type I genes (*COL1A1* and *COL1A2)*, responsible for mild to severe osteogenesis imperfecta that can be revealed in adulthood [34]. Moreover, other rare mutations, reported in young osteoporotic adults, are regarded as extracellular matrix genes or cell factors involved in the regulation of bone remodeling. Quite recently, heterozygous Wnt family member 1 (*WNT1*) mutations have been found in adults with autosomal dominant early-onset osteoporosis, overlapping in some aspects with the same mutation (in this case involving both alleles), and causing severe osteogenesis imperfecta [35]. 

The low-density lipoprotein (LDL) receptor-related protein 5 (*LRP5*) gene, whose inactivating mutations have been associated to low bone mass in osteoporosis pseudoglioma syndrome, can be considered to be another strong candidate contributing to idiopathic osteoporosis. LRP5 protein, a member of the LDL receptor superfamily, acts as a co-receptor that is indispensable for activating the Wnt-pathway and regulating bone formation. Mutation in the *LRP5* gene was firstly identified in osteoporosis pseudoglioma syndrome, an autosomal recessive disease characterized by severe osteoporosis revealing in infancy and associating with a congenital blindness. Lately, some polymorphic *LRP5* variants were also consistently associated with BMD and fracture risk (*p.Val667Met* and *p.Ala1330Val*) in association studies, and very few novel heterozygous pathogenic mutations with a major effect were identified in children and adolescents with juvenile idiopathic osteoporosis. Overall, these studies showed that either rare (e.g., mutations) or more common (e.g., polymorphisms) genetic variants of *LRP5* are associated with a wide spectrum of phenotypes characterized by early or maturity-onset bone fragility in both genders [36]. 

## 4. Epidemiology of Osteoporosis and Fractures

Osteoporosis in males represents a pathological condition that remains largely underdiagnosed and undertreated mainly due to the low frequency of screening and controversies in BMD testing standards [37,38]. Based on the WHO diagnostic criteria, it has been estimated that about 10–25% of the male “Medicare population” could suffer from osteoporosis [39,40]. A European report based on a collaboration between the International Osteoporosis Foundation (IOF) and the European Federation of Pharmaceutical Industry Associations (EFPIA) confirmed both that over 6 million men had osteoporosis in 2010 according to densitometric criteria and that the prevalence of this condition significantly increases with ageing [41]. 

However, the rate of osteoporosis in men is underestimated, as many subjects who experienced fragility fractures do not report a BMD impairment [39]. In general, the incidence of fractures in men has a bimodal peak at 18–45 years (traumatic fractures) and at 75–80 years of age (osteoporotic fractures) [42].

Although women are characterized by a significantly higher prevalence of osteoporosis, about 30–40% of overall osteoporotic fractures belong to men [43]. In particular, in US and Europe, the incidence of hip fractures ranges from 0.5 per 1000 patients per year at the age of 60 up to 13 per 1000 patients per year by the age of 85 [44,45]. While Caucasian women have a hip fracture incidence four-fold higher than men, Asian women have the same incidence of hip fracture compared to men [3]. It is important to underline that a significant proportion of men who suffer hip fractures have broken other bones before breaking their hip [46]. In particular, after the first hip fracture, the risk of a subsequent hip fracture increases about five-fold, and it has been estimated that about 27% of men who had suffered a hip fracture sustained subsequent fractures in their remaining lifetime [47,48].

It is also well recognized that hip fracture in men is strictly associated with an increased morbidity and mortality. Indeed, Diamantopoulos et al. have reported that males’ mortality rates after hip fracture are about three-fold higher than females [45].

## 5. Risk Factors and Risk Estimation

Osteoporosis screening in older men has been proposed because it is an accepted strategy in older women. Some guidelines recommend BMD testing in all men aged 70 years or older and in those aged 50–69 years with risk factors [49,50]. The U.S. Preventive Services Task Force made no recommendation about osteoporosis screening in men [51]. A study that compared proposed strategies for selecting men aged 70 years or older for osteoporosis screening reported that the Osteoporosis Self-Assessment Tool (OST), which relies on age and weight only, performed slightly better than the more complex strategies [52]. The risk factors for osteoporosis and fracture in men are almost the same as in women. Thus, the presence of risk factors (e.g., family history, long-term glucocorticoid therapy, hypogonadism, low body weight, low bone mineral density, family history of hip fracture, cigarette smoking, excessive alcohol consumption, low dietary calcium intake, vitamin D deficiency, neuromuscular disorders, long-term immobilization) should be particularly considered in order to identify men with osteoporosis [50,53]. Secondary osteoporosis is common in men, and it is likely that the majority of men between the ages of 50 and 70 with osteoporosis and fragility fractures have secondary causes [54]. In the prospective long-term observational study of the MrOS cohort, which enrolled almost 6000 men in United States, several risk factors, such as, among other things, older age (≥75 years), low hip BMD, smoking, and history of fracture, were associated with an increased risk of hip fracture [55]. It should be noted that there are frequent overlaps due to the pathophysiological links between what can be considered a risk factor for primary osteoporosis and a cause for secondary osteoporosis (e.g., smoking, chronic obstructive pulmonary disease, gluococorticoid use, sarcopenia). 

In older adults, both an increased prevalence of sarcopenia and its association with falls and fragility fractures have been reported. The SARC-F, a questionnaire developed for rapidly assessing the presence of sarcopenia and estimating the fall risk, was found to be predictive of hip fractures independently of the FRAX score (adjusted Hazard Ratio, HR: 1.24 and 1.15 in men and women, respectively) [56]. In keeping with these findings, prevalent single or multiple falls were found to be associated with incidental major osteoporotic fractures (MOF) independent of FRAX (with or without BMD) in men, but not in women [57]. Moreover, in the first year after a single fall, men had an increased risk of fracture (FRAX-adjusted HR 3.47), which remained elevated up to 10 years among individuals with two or more recurrent falls [57]. It is known that, when individually taken, most clinical risk factors have a weak effect on fracture risk prediction, while their importance becomes evident when the risk factors are combined in a composite score. For this reason, several fracture risk prediction tools have been developed, including the FRAX, the Garvan fracture risk calculator, and the QFracture score [58,59]. However, the predictive value for estimating the fragility fracture risk of these algorithms has not been studied in men as extensively as in women. The FRAX tool, which estimates the fragility fracture risk over 10 years, was found to be strongly predictive of MOF and hip fracture in both women and men without significant sex interaction, regardless of the inclusion of BMD data [60]. In men at 65 years of age and older, Gourlay et al. compared the fracture risk assessment algorithms for predicting incident fractures. The QFracture, FRAX, Garvan, and femoral neck BMD T-score performed similarly in terms of the discriminative ability to identify men with hip fractures (accuracy ranging from 0.77 up to 0.79 for all tools) [61]. Although falls and impaired muscle function (sarcopenia) are predictors of incident fractures, falls are not included in the FRAX score. However, the fall propensity is incorporated into the other risk calculators, as an indirect measure of muscle function, but muscle strength is not directly included in any of the tools. This seems to be of importance considering that, in metanalysis studies, past falls were predictive of incident fractures regardless of the FRAX score and that, in older men, muscle mass, strength, and function predicted fractures independent of the FRAX score, even after adjustment for BMD, with the exception of the appendicular lean mass [62]. Another important variable that may affect the fragility fracture risk in men is the presence of type 2 diabetes, which is associated with an increased fracture risk independent of the FRAX score. Thus, for improving the accuracy of FRAX in diabetic subjects, the use of the rheumatoid arthritis input, and, for the trabecular bone score (TBS) adjustment, a decrease in the femoral neck T-score input by 0.5 SD, and an increase in age input by 10 years have been proposed [63]. 

The efforts in identifying subjects at risk for fracture is of crucial importance in clinical practice, and it is mandatory to progressively improve our ability in this field. As compared to women, men experience fractures about 10 years later in life and, due to their more advanced age, they may suffer for more comorbidities than women, thus explaining the two-fold increased mortality in men than in women. Thus, while the fracture risk is lower in men than in women, the consequences are greater, and therefore identifying and treating men at risk for fracture could impact on mortality more than in women.

## 6. Bone Mineral Density Testing

The concept of osteoporosis is intimately linked to the risk of fracture, as fractures often occur in subjects with low BMD [64]. After a “low energy” fracture, the absolute risk of refracture for all skeletal sites is similar for both males and females; however, given the low initial prevalence of fractures in the male population, the relative risk is higher in men than in women [65].

**Figure 1:**
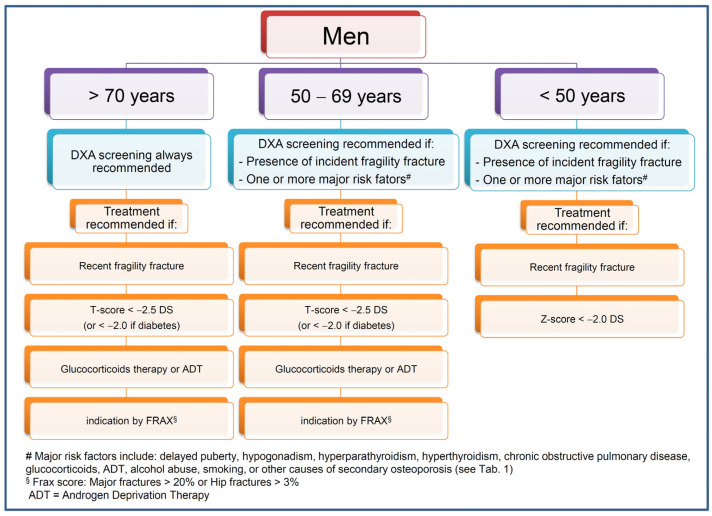
Proposed indications for DXA screening and treatment in men.

The measurement of BMD has been shown to be cost-effective for the female population older than 65 years and for younger postmenopausal women with risk factors [66]. Similarly, BMD testing in men over 70 years is widely recommended, as ageing is considered to be an overriding risk factor for fracture in this population [50,64,67]. Men between 50 and 69 years should be tested if they have one of the following risk factors: history of diseases/conditions such as hypogonadism, delayed puberty, hyperparathyroidism, hyperthyroidism, rheumatoid arthritis, or chronic obstructive pulmonary disease; drugs such as glucocorticoids or gonadotropin-releasing hormone agonists, lifestyle habits such as alcohol abuse or smoking [50] (Figure 1). Some authors have also suggested screening men who have hypercalciuria and/or nephrolithiasis and those who have underwent a bariatric surgery procedure [54,68].

The diagnosis of osteoporosis in men can also be done on a clinical basis, that is the occurrence of spontaneous or low-trauma fractures (i.e., a fall from a standing height). In this very case, the measurement of BMD becomes important not for the diagnosis of osteoporosis, but for both determining the degree of bone loss and for following up on the effects of the therapies.

Bone sites where DXA can be performed are the lumbar spine (L1–L4 on antero-posterior projection), the hip (femoral neck or total proximal femur), and the forearm (33% radius of non-dominant forearm), if the hip and/or spine cannot be measured or interpreted. Forearm BMD should be measured in the presence of hyperparathyroidism or in very obese patients, whose weight go beyond the limit for the DXA table. Recently, the International Society for Clinical Densitometry (ISCD) reported that TBS is associated with hip and major osteoporotic fracture risk in men over the age of 50 years [69].

It is commonly accepted that low BMD is a very good indicator for selecting men who need pharmacological treatment, particularly because the lower is BMD, the higher is the efficacy of therapies [70]. Although the T-score (the number of standard deviation units in relation to the young reference healthy population) represents a strong predictor of fracture in men, the method to calculate it has been debated. Osteoporosis is identified by values lower than –2.5 SD, both for females and males [71], but the T-score may vary depending on whether it is calculated on the basis of female or male BMD reference ranges. Moreover, in individuals younger than 50 years of age, the Z-score (the number of standard deviation units in relation to the age-matched reference healthy population) is considered to be the best parameter for evaluating the fracture risk [71].

The National Osteoporosis Foundation (NOF), the Endocrine Society, as well as the Italian Society for Osteoporosis, Mineral Metabolism, and Bone Diseases (SIOMMMS) recommend the use of a male-specific reference range [49,50,72], while the ISCD and the International Osteoporosis Foundation (IOF) recommend the calculation of the T-score in men on the basis of a young female normative database [69,73].

Roughly, each SD reduction in the T-score value doubles the risk of fracture both in males and females, as far as BMD and fracture risk show an overlapping trend in the two sexes. As a matter of fact, if a T-score of –2.5 SD identifies a high relative risk of fracture for women using a female reference range, it is reasonable that the same relative risk will be found at the same T-score threshold for men using a males-derived reference range. The suggestion for using sex-specific reference database rests on this assumption [30].

On the other hand, the argument for using a female-derived reference database is that men have a risk of fracture that is generally lower than women. Therefore, the same T-score threshold will give back an identical absolute risk of fracture in males and females only if it is calculated on the same reference range population (i.e., 25–30-year-old young women). However, as men have bigger bones than women, by using a female reference range, we would underestimate the rate of osteoporosis in men. In this very case, as the value of BMD, rather than T-score, is the real determinant of absolute fracture risk, a lot of men would not be offered a proper treatment because they are not classified as having osteoporosis [30].

In conclusion, the use of a male-based reference database appears to be more appropriate and it allows treating a higher number of patients consistently with epidemiological data on fracture incidence in men [74,75,76,77]. 

## 7. Laboratory Testing for Differential Diagnosis

Secondary osteoporosis is reported to be more frequent in men than in women, rating up to 60% of all the cases (Table 1). As mentioned above, the most common causes of male osteoporosis are corticosteroids use, unhealthy lifestyle (i.e., smoking and/or high alcohol consumption), primary or secondary hypogonadism, vitamin D deficiency, and low calcium intake. Albeit less frequently, other diseases or conditions may cause osteoporosis in men [78]. Therefore, it is important to rule out all of these possible causes, even though the cost-effectiveness of laboratory testing for evaluating osteoporosis in men is still unclear [79].

In all the men diagnosed with osteoporosis, both a complete history and physical examination should be done in order to find clues of possible secondary osteoporosis.

A targeted laboratory assessment based on history and a physical exam will allow for the diagnosis of the presence of secondary causes. The measuring of complete blood count, alkaline phosphatase, liver function, serum calcium, phosphate, creatinine, estimated glomerular filtration rate, 25-hydroxyvitamin D [25(OH)D], total testosterone, and 24-h urinary calcium excretion should be considered. According to the history of the patients and/or to specific biochemical-clinical or phenotypic aspects, testing should include index exams for the suspected disease, such as calculated free testosterone, luteinizing hormone (LH), serum protein electrophoresis, tissue transglutaminase antibodies, PTH, or TSH [50]. Other guidelines have highlighted the need of ruling out Cushing syndrome by a 24-h urinary cortisol and/or dexamethasone suppression test [72].

A recent study has shown that most recommended laboratory tests prescribed to assess and achieve a differential diagnosis of metabolic bone diseases are unlikely in older men, except perhaps for vitamin D and alkaline phosphatase dosages [80]. 

Although more evidence-based data are needed in order to validate the role of laboratory tests, we must be aware that male osteoporosis is frequently due to other conditions that must be diagnosed and cured. A correct laboratory approach before stating anti-osteoporosis therapies should be considered as a cornerstone in the management of osteoporosis in men [81]. 

## 8. Pharmacotherapies of Osteoporosis in Men

Gendered medicine represents an important concept in order to appropriately set up the most effective, patient-centered, treatment option. Unfortunately, since most of the randomize controlled trials (RCTs) for anti-fracture drug development have been performed in post-menopausal women, currently, there is not a universally shared and validated strategy for therapeutic decision-making in men [3,50]. In fact, most of the RCTs performed in men considered the change in BMD as a primary end point, since they were not powered to assess the reduction in fracture risk. The overall results from these RCTs exhibited effects on BMD, bone turnover, and trends in fracture reduction similar to those reported in larger registration RCTs in postmenopausal women [82,83,84]. Indeed, it should always be taken into consideration that up to 60% of male subjects with a diagnosis of osteoporosis may suffer from a secondary form, and that the correction of the secondary cause must always be pursued.

Based on a cost-effectiveness analysis by the NOF, subsequently endorsed by the Endocrine Society, the following treatment indications have been recommended for men: (a) presence of hip or vertebral fracture without major trauma; (b) subjects without fragility fractures but showing lumbar spine and/or hip BMD −2.5 SD below the mean of normal young males; (c) individuals undergoing long-term glucocorticoid therapy with >7.5 mg/dL daily of prednisone or equivalent for more than 3 months; (d) diagnosis of osteopenia in men whose lumbar spinal and/or hip BMD T-score values range between −1.0 and −2.5 SD with a FRAX risk-calculated 10-year fracture probability equal or greater than 3 and 20%, respectively, for hip and major osteoporotic fractures, although this approach may underestimate the fracture risk [50,81,85]. 

As reported in studies in post-menopausal osteoporotic women, where the use of antiresorptive/anabolic drugs lacking in adequate calcium and vitamin D intake is not evidence based, calcium and vitamin D supplementation should also be considered in men as a fundamental part of all pharmacological treatments of osteoporosis [3,50,81,84]. Bone active agents currently approved for the pharmacological therapy of male osteoporosis consist of antiresorptive agents, such as aminobisphosphonates (NBPs) or denosumab and the osteoanabolic agent teriparatide (Table 2).

### 8.1. Antiresorptive Agents

This class of drugs mainly includes oral NBPs (alendronate, risedronate, and ibandronate), intravenous NBPs (zoledronate and ibandronate), and denosumab. 

#### 8.1.1. Amino-Bisphosphonates (NBPs)

Either oral alendronate or risedronate were demonstrated to be effective in increasing BMD and decrease bone turnover markers in osteoporotic men [90,91,92,93,94]. As mentioned above, these clinical studies were not designed or powered to assess anti-fracture efficacy. However, subsequent meta-analyses of these RCTs data evidenced that alendronate is effective in reducing the risk of vertebral fractures in men with low bone mass or fractures, but there is no sufficient evidence demonstrating a significant effect on non-vertebral fractures [86]. Likewise, open-label studies with risedronate revealed that it reduces the incidence of a new vertebral fracture by 60% and of non-vertebral fractures by 47% in men with primary or secondary osteoporosis versus placebo [87]. Currently, different oral formulations of alendronate (10 mg daily or 70 mg weekly) or risedronate (5 mg daily, 35 mg weekly, or 150 mg monthly) are available on the market. A single RCT assessed the efficacy of oral ibandronate (150 mg once-monthly) in 132 men with primary, idiopathic, or hypogonadism-related low bone density [95]. At 1 year of treatment, ibandronate significantly increased BMD at the lumbar spine or hip and significantly reduced bone turnover marker levels as compared with placebo.

A more recent RCT, designed with fracture end points, has been performed in order to assess the efficacy of intravenous zoledronate (yearly 5 mg infusion) on the prevention of fractures in a large cohort of 1199 men aged 50 years or older, with primary or hypogonadism-associated osteoporosis [96]. After 2 years, zoledronate-treated patients had a significant 67% reduction in the relative risk of one or more new morphometric vertebral fractures versus the placebo group. Interestingly, the rates of nonvertebral fracture were also consistently lower with zoledronate than with placebo, showing similar point estimates to those reported in larger studies on women. Moreover, the effects of therapy on BMD, bone turnover markers, and the reduction in vertebral fracture risk were shown to be similar to the results of the pivotal trials in postmenopausal women, further supporting the fact that this agent is effective in both genders. Indeed, the same conclusion had been reached the year before by a gender-specific analysis of the HORIZON RFT trial [97].

Overall, either oral or intravenous NBPs have been demonstrated to be generally well tolerated in osteoporotic males, with adverse events similar both in type (upper gastrointestinal symptoms with oral compounds and acute phase reaction in up to 40% of cases with the first zoledronate infusion) and occurrence rate, as observed in females. Other complications associated with long-term NBPs treatment regimens such as osteonecrosis of the jaw and atypical femoral fractures were shown to be very rare.

#### 8.1.2. Denosumab

Denosumab, the monoclonal antibody against the receptor activator of nuclear factor–kB ligand (RANKL), has a well-documented antiresorptive activity on bone. Following the positive results of RCTs in postmenopausal women [84], an RCT in men was firstly directed at subjects receiving androgen deprivation therapy (ADT) for non-metastatic prostate cancer [88]. In these subjects, the standard dosing regimen of subcutaneous 60 mg denosumab, every 6 months, reduced bone turnover, increased BMD, and significantly reduced the incidence of new vertebral fractures at 36 months. Interestingly, always in prostate cancer patients, a subsequent trial using higher denosumab dosages of 120 mg every 6 months demonstrated a delay in the occurrence of bone metastasis [98]. 

The results emerging from a subsequent 12-month RCT in men with low BMD, showing significant increases in both lumbar and femoral BMD over placebo (5.7% and 2.4% increases at 12 months, respectively), caused the U.S. Food and Drug Administration (FDA) and European Medicines Agency (EMA) to extend the use of denosumab (60 mg subcutaneously, every 6 months) to all male subjects at a high risk of fracture [99]. As for zoledronate, the effects of denosumab on BMD were similar to those reported in the larger RCTs in postmenopausal women with osteoporosis, where efficacy in the prevention of vertebral and non-vertebral fractures was also demonstrated. Likewise, as reported for NBPs, for denosumab, the incidence of adverse events was similar to that reported in women. These mainly include increases risk of eczema and cellulites in the first years of treatment. Moreover, long-term denosumab treatment has also been associated with jaw osteonecrosis and atypical femoral fractures. Recently, some concerns have been raised for the possible rebound of bone turnover and the associated risk of multiple vertebral fractures after denosumab discontinuation, but while this has been rarely described in women (particularly in case of a prevalent vertebral fracture before denosumab initiation) [100], the corresponding information in men is lacking. 

### 8.2. Bone Anabolic Agents

Despite this, different bone anabolic agents (teriparatide, abaloparatide, and romosozumab) are actually approved in most countries for the treatment of postmenopausal osteoporosis; the only available osteoanabolic agent approved worldwide for the treatment of osteoporosis in men is teriparatide, the 1–34 amino terminal fragment of the intact PTH molecule. The therapeutic efficacy of this drug in men has been demonstrated in different clinical trials [89,101,102,103]. 

A significant increase in vertebral BMD in middle-aged men with primary osteoporosis was observed in a pilot study using daily subcutaneous injections of teriparatide for 12 months [103]. Subsequently, a small study was specifically designed to address the efficacy of teriparatide in 23 middle-aged men with idiopathic osteoporosis and markedly reduced bone formation indexes at histomorphometry, 78% of whom had sustained fragility fractures [101]. After 18 months, active treatment with teriparatide (400 IU daily subcutaneous injections) was associated with important increases in both lumbar spine and hip BMD, together with increases in bone turnover markers. In a larger, placebo-controlled RCT, performed on a mixed sample of 437 men with idiopathic osteoporosis, age-related osteoporosis, or osteoporosis secondary to hypogonadism, two different teriparatide dosages (20 and 40 mcgs/day) were compared versus placebo [102]. Albeit the study was stopped at 11 months (because of a finding of osteosarcomas in rats in routine toxicology studies), a significant increase in BMD was observed in both active treatment groups over placebo (5.9% with 20 mcgs and 9.0% with 40 mcgs at the lumbar spine, and 1.5% and 2.9%, with 20 mcgs with 40 mcgs, at the hip, respectively). Consistent with the anabolic activity of this drug, bone formation markers significantly increased during treatment, with a greater extent than bone resorption markers. The response to teriparatide was to be similar and independent from gonadal status, age, baseline BMD, and body mass index. Adverse events such as nausea, headache, and dizziness were similar in the placebo and 20-mcg groups, while they were more frequent in the 40-mcg group. 

As for oral NBPs, these trials were not designed to have fractures as an end point, but an indication for a reduction in vertebral fracture incidence has emerged in the 18-month follow-up analysis of the latter RCT, with a 51% risk reduction, close to statistical significance, in those subjects previously treated with teriparatide [89]. In a subsequent trial in glucocorticoid-induced osteoporosis, including both sexes, teriparatide treatment revealed to be superior to alendronate in increasing BMD and preventing fractures in the overall cohort [104]. However, the male-specific analysis, likely due to the limited sample of males, did not to achieve statistically significant differences [105].

Albeit current evidence suggests that NBPs or denosumab should be recommended as first-line pharmacotherapy in osteoporotic men [50], from at least a theoretical point of view, osteoanabolic teriparatide regimens would represent a more logical approach in men with idiopathic osteoporosis and low bone turnover [30]. This is also the case for some forms of secondary osteoporosis, such as glucocorticoid-induced osteoporosis [104,105] or in type 2 diabetes osteopathy [106], whose pathogenic mechanisms are mainly linked to impaired bone formation.

### 8.3. Bone Anabolic Agents Testosterone Replacement

Testosterone replacement therapy (TRT) may be considered in patients with hypogonadism, which is a major cause of secondary osteoporosis in men. As any replacement therapy, there is no role for testosterone therapy in eugonadal men. Although men with the lowest serum T concentrations have been shown to achieve the greatest increase in BMD, scarce data are available on fracture risk [107]. Notwithstanding the well-known positive effect of androgens on periosteal bone, TRT has only a moderate effect on lumbar BMD (≈2% increase) and a scarce effect on femur BMD [108].

The possible negative effect of T on the risk of prostate cancer and cardiovascular events in elderly patients is still a matter of debate and, therefore, the risk/benefit ratio of a long-term TRT in these individuals is still unknown [109]. Indeed, a recent case-crossover study comparing 6-month T use for 39,622 subjects concluded that men without cancer prescribed testosterone therapy had approximately twice the risk of venous thromboembolism within the 1-, 3-, and 6-month case periods compared with the equivalent control periods 6 months earlier, with some evidence that the association was more pronounced among younger men [110].

Importantly, the antiresorptive and osteoanabolic agents available in men have been demonstrated to be effective in preventing BMD decrease even in hypogonadal patients. As a consequence, the Endocrine Society’s recommendations suggest the use of bisphosphonates and other approved therapies for hypogonadal men [50]. The possible use of bone active therapy may be considered even in hypogonadal men at high risk of fracture, who are already treated with T for hypogonadal symptoms. On the other hand, using androgen replacement as ‘bone drug’ should be considered in hypogonadal men (i.e., T levels <200 ng/dL) with hypogonadal symptoms (i.e., low libido, hot flushes, unexplained chronic fatigue who are symptomatic), and in whom the approved pharmacological agents for male osteoporosis cannot be used due to contraindications. 

## 9. Future Treatments

### 9.1. Selective Estrogen Receptor Modulators (SERMs) and Selective Androgen Receptor Modulators (SARMs)

The action of estrogens on the male skeleton (i.e., achievement of peak bone mass, regulation of bone turnover and prevention of bone loss) has suggested the administration of drugs with an estrogenic action on bone, such as SERMs, also in men, in order to prevent bone loss [3]. SERMs administered in males with low estrogen levels reduced bone resorption markers [111], and, in hypogonadal males under androgen deprivation therapy for prostate cancer, they prevented bone loss and halved the risk of vertebral fractures in respect to placebo [112]. 

The action of androgens on bone is mainly due to the enhancement of periosteal apposition, to the anabolic effect on muscle mass, and to their peripheral conversion into E. From a theoretical point of view, the SARMs may represent an ideal drug, as, by allowing a tissue-selective interaction with the androgen receptor, they can exert an optimal effect on bones and muscles, with less involvement of other tissues such as prostate, heart, and kidney [3]. The studies with SARMs, however, did not show particular advantages, while some toxic effects were reported [3]. 

#### 9.1.1. New Antiresorptive Drugs

The bone resorption process is characterized by three phases: Adhesion of osteoclasts to the bone surface in order to have the resorption lacuna; Acidification of the resorption lacuna in order to dissolve the mineral component of bone; Degradation of the bone matrix through the release of proteolytic enzymes [113]. Cathepsin K (CK) is a lysosomal protease released by active OCs (osteoclasts) during bone resorption for the degradation of the organic matrix. As CK does not affect the production of OB-stimulating factors, it is reasonable that CK-inhibitors may exert a lower inhibition of bone formation than N-BPs or denosumab [114]. Odanacatib was the first CK-inhibitor used in clinical studies on osteoporosis, and it showed a good efficacy in suppressing bone resorption markers, in increasing BMD, and in reducing fracture risk, together with a lower inhibition of bone formation. Unfortunately, an unexpectedly increased risk of stroke stopped the clinical development of Odanacatib, but there are other CK-inhibitors that are under study. 

Vacuolar H^+^-ATPases (V-ATPases) of the OC ruffled border are essential for the acidification of the resorption lacunae and for the dissolution of the bone mineral, thus allowing proteases digestion of the bone matrix protein. Although V-ATPases are ubiquitous proton pumps, some drugs targeting osteoclast-ruffled border V-ATPases have been identified [115], and they showed an ability to prevent bone loss in ovariectomized mice [116].

Src proto-oncogene is a member of the tyrosine kinases, which are highly expressed in OCs. Src proto-oncogene contributes to the OCs’ survival and to the development of their ruffled border. In animal models, the Src proto-oncogene inactivation causes increased BMD and osteopetrosis [117]. The reduction in Src proto-oncogen expression has been suggested to increase OB differentiation and bone formation [118]. Saracatinib is a competitive inhibitor of Src kinase. This agent induces the in vitro inhibition of OC formation and activity. In addition, in a phase I trial in healthy men, saracatinib administration led to a dose-dependent reduction in bone resorption markers, without both significant effect on bone formation markers and serious adverse events [119].

#### 9.1.2. New Anabolic Drugs

Drugs counteracting physiological antagonists (i.e., sclerostin, dickkopf-1 (DKK-1), secreted frizzled-related proteins) of the Wnt–β-catenin signaling pathway are studied as targets for new anabolic agents. 

For the coupling mechanisms between bone formation and resorption, the antiresorptive therapy with suppression of osteoclast (OC) activity is followed by a subsequent reduction in bone formation, while the anabolic therapy with an activation of osteoblast (OB) activity is followed by a subsequent increase in bone resorption, the latter limiting the anabolic window of treatment with anabolic drugs such as teriparatide or abaloparatide. In the last years, some molecules that act in uncoupling bone formation and resorption, thus having a combined anabolic and antiresorptive effect on bone, have been studied, and, among these romosozumab, a monoclonal antibody against sclerostin has been marketed at least in some countries for the treatment of postmenopausal osteoporosis. In a phase III randomized placebo-controlled trial in men, the treatment with romosozumab induced a significantly higher increase in spine and hip BMD than placebo, over a period of 12 months [120]. Cardiovascular adverse events with romosozumab treatment have raised concerns regarding the safety of this drug, therefore a deep re-evaluation of the data is ongoing.

Apart from romosozumab, other monoclonal antibodies against DKK-1 have been developed [121]. Recently, a bispecific heterodimeric antibody targeting both sclerostin and DKK1 have been created and, in animal models, it has induced larger increases in bone mass and bone strength than with either romosozumab or DKK1-Ab treatment alone [122].

Sirtuins (Sirt) are considered to be the master regulators of several cellular processes, and mice with Sirt1 deletion in osteoprogenitor cells suffer from an impaired bone formation and consequentially reduced cortical bone mass [123]. Sirt1 seems to be a positive regulator of bone formation and resveratrol; moreover, a Sirt1 activator induced an increase in bone formation markers and bone mass in elderly obese men [124].

Nitric Oxide (NO), through the activation of soluble guanylate cyclase (sGC), promotes OB proliferation and survival. Cinaciguat, a sGC activator, increased bone formation and improved indices of bone microarchitecture in animal models [125]. Insulin, via the NO/sGC pathway, favors OB proliferation and survival, and, accordingly, in type 1 diabetes, bone damage seems to be due to a reduction in bone formation [126]. In animal models with type 1 diabetes, treatment with cinaciguat, increased OB proliferation, and survival with a final increase in bone mass [127].

#### 9.1.3. New Drugs with Dual Action, Anabolic, and Antiresorptive Activity

Activin A is a protein of the extracellular bone matrix and belongs to the TGFβ/BMP superfamily. It plays a role in osteoclastogenesis and also negatively regulates OB differentiation. Serum levels of activin A are higher in postmenopausal women with osteoporosis in respect to women without osteoporosis [128]. Activin A antagonists have been suggested for having a dual anabolic–antiresorptive effect on bone [129]. Sotatercept, an activin A antagonist, increased bone formation and reduced bone resorption in primates [130], and, in healthy postmenopausal women, it induced an increase in bone formation, together with a reduction in bone resorption markers [131]. 

Semaphorins regulate cell–cell interactions, cell adhesion, and motility in several tissues, and, in the bone, semaphorin signaling is involved in the communication between cells such as OBs and OCs. Semaphorin 3A, secreted by OBs and OCs, stimulates bone formation and inhibits bone resorption, as shown in animal models treated with Semaphorin 3A [132]. On the contrary, semaphorin 4D, secreted by OCs, which reduces bone formation and increases bone resorption and bone loss, was prevented in animal models treated with antisemaphorin 4D antibodies [133]. Agonists of semaphorin 3A and antagonists of semaphorin 4D should be investigated as drugs for osteoporosis. 

Hydrogen sulfide (H2S) has several physiological effects in different tissues, and, in cellular and animal models, it inhibits OC activity and, consequently, bone loss and stimulates osteoblastic differentiation, thus increasing bone formation [134]. A drug combining alendronate, which has a high affinity for bone matrix, with H2S-releasing substances has been developed, thus combining the bone anticatabolic and anabolic functions. The preliminary cellular data are interesting [135]. 

The kynurenine (Kyn) pathway, which is part of the tryptophan degradation pathway, seems to play a role in the osteogenic differentiation and osteoclastic activity [136]. Blocking this pathway in cellular and animal models reduced OB differentiation and increased OC number with consequent osteoporosis [137], while administering picolinic acid, the end-product of the Kyn pathway, determined a strong osteogenic effect with increased bone formation [138]. Substances interfering with tryptophan metabolites could be developed as drugs with a dual antiresorptive and anabolic action on bone. 

## 10. The Management of Osteoporosis Men

The proposed indications for DXA screening and treatment in men are summarized in Figure 1. While the BMD determination by DXA is recommended for all men above 70 years of age, in younger individuals, the screening for osteoporosis is recommended in the presence of incident fragility, fractures, and/or of one major risk factor among delayed puberty, hypogonadism, hyperparathyroidism, hyperthyroidism, chronic obstructive pulmonary disease, use of glucocorticoids or ADT, alcohol abuse and smoking habit, or other causes of secondary osteoporosis (Table 1). Treatment with bone active drugs is always recommended in the presence of a recent fragility fracture. In non-fractured patients above 50 years of age, an anti-fracture treatment is recommended in the presence of a BMD T-score below −2.5 or below −2.0 in diabetic patients, or in the presence of glucocorticoid therapy, ADT, a risk of major osteoporotic fractures above 20%, or of hip fractures above 3%, as evaluated by Frax score. In non-fractured patients below 50 years of age, an anti-fracture treatment is recommended in the presence of a BMD Z-score below −2.0.

Nowadays, male osteoporosis is still underestimated and underdiagnosed with the logical consequence that, often, men are not offered an adequate treatment. This lack of adequate management of osteoporosis in men may have an impact on the health-related life quality (HRQoL) in these patients. A recent systematic review and meta-analysis (of 14 and 10 studies, respectively) showed that men with osteoporosis had a lower HRQoL than men without osteoporosis, with hip fracture, vertebral fractures, or wrist fractures severely impairing HRQoL of men. Interestingly, BMD at spine and femur was positively correlated with HRQoL, and effective anti-osteoporotic drugs could improve the HRQoL of men [139].

The strengths of the present study are firstly related to the fact that it includes not only the available evidence regarding the risk factors and the crucial elements for diagnosing and treating osteoporosis in men, but also some potential recommendations regarding how to manage this condition in a real life setting. Secondly, the present review sheds light on the future possibilities for the treatment of osteoporosis in men.

However, this study has the limitation of being a narrative review rather than a systematic review, and, consequently, a selection study and information bias could not be excluded. Accordingly, the proposed flow-chart on the management of osteoporosis in men has to be considered an expert position, rather than evidence-based guidelines, since the Grading of Recommendations Assessment, Development and Evaluation (GRADE) system has not been adopted. Consequently, this study does not aim to change the medical practice on the basis of literature evidence. 

## 11. Conclusions

Notwithstanding the several weaknesses of the data regarding the pharmacological prevention of fractures in men, some of the available therapies may exert an important effect in males as well as they do in females. Nevertheless, as fractured men have a higher morbidity and mortality than fractured women, a prompt diagnosis and a consequent treatment appear to be crucial in this population. 

Recent studies, both basic and translational, have paved the way for new acquisitions in the pathophysiology of skeletal metabolism, identifying or suggesting a role of “new” molecules and “new” molecular pathways for bone health, both in females and in males. There are hopes for future therapeutic agents that will reduce bone fragility and fracture risk with the possible result of decreasing mortality in men with osteoporosis.

## Figures and Tables

**Table 1:** Conditions described as inducing osteoporosis in men.

**Medications**
Long term glucocorticoid therapy
GnRH agonists or analogs
Cytotoxic agents
Anticonvulsants
Excessive thyroxine doses
Heparin
Immunosuppressive agents (cyclosporine)
Antiretroviral therapy for HIV
Use of tricyclic antidepressants
**Diseases**
Endocrine
Hypogonadism, Hyperparathyroidism, Cushing’s syndrome, Type 1 and type 2 diabetes, Hyperthyroidism, Acromegaly, GH deficiency, Delayed puberty
Chronic liver diseases, Inflammatory bowel disease, Celiac disease
Rheumatologic
Rheumatoid arthritis, Systemic lupus erythematosus, Systemic sclerosis, Ankylosing spondylitis
Hematologic
Lymphoma and Leukemia, Multiple myeloma, Systemic mastocytosis
Renal
Chronic renal failure, Renal tubular acidosis, Idiopathic hypercalciuria, Nephrolithiasis
Pulmonary
Chronic obstructive pulmonary disease (COPD)
Neurologic
Parkinson’s disease, Neuromuscular disorders
Genetic
Hypophosphatasia, Osteogenesis Imperfecta, Cystic fibrosis, Thalassemia
Other
Organs transplantation, Hemochromatosis, HIV infection, Bariatric surgery procedures
**Lifestyle Habits**
Low calcium intake and/or Low protein intake
Sedentary lifestyle
Cigarette smoking
Heavy alcohol consumption
High caffeine intake
**Others**
Ageing
Family history of osteoporosis or fracture in first-degree relatives
Personal history of fracture as an adult
Low bone mineral density
Vitamin D deficiency
Low BMI (<18 kg/m^2^)
Long-term immobilization and/or Decreased mobility and/or Sarcopenia

**Table 2:** Currently approved drugs for the treatment of osteoporosis in men.

Drug	Administration Route Dose	Effect on BMD	Fracture Risk Reduction in Specifically Designed RTCs		
			Vertebral	Non-Vertebral	Hip
Alendronate	Oral 10 mg/day 70 mg/week	Yes	No (Yes) (only in a meta-analysis of RCTS) [86]	No	No
Risedronate	Oral 5 mg/day 35 mg/week 75 mg/twice a month	Yes	No (Yes) (only in an open-label study) [87]	No (Yes) (only in an open-label study) [87]	No
Ibandronate	Oral 150 mg/month	Yes	No	No	No
Zoledronate	Intravenous injection 5 mg/year	Yes	Yes	No	No
Denosumab	Subcutaneous injection 60 mg/every 6 months	Yes	No (Yes) (only in men receiving androgen deprivation therapy for non-metastating prostate cancer) [88]	No	No
Teriparatide	Subcutaneous injection 20 μg/day	Yes	No (Yes) (follow-up analysis of a RCT, with a risk reduction close to statistical significance) [89]	No	No

## References

[1] Melton L.J., Chrischilles E.A., Cooper C., Lane A.W., Riggs B.L. (1992). Perspective. How many women have osteoporosis?. J. Bone Miner. Res..

[2] Harvey N.C.W., McCloskey E.V., Mitchell P.J., Dawson-Hughes B., Pierroz D.D., Reginster J.-Y., Rizzoli R., Cooper C., Kanis J.A. (2017). Mind the (treatment) gap: A global perspective on current and future strategies for prevention of fragility fractures. Osteoporos. Int..

[3] Gennari L., Bilezikian J.P. (2018). New and developing pharmacotherapy for osteoporosis in men. Expert Opin. Pharmacother..

[4] Seeman E. (2003). Periosteal bone formation—A neglected determinant of bone strength. N. Engl. J. Med..

[5] Sinnesael M., Boonen S., Claessens F., Gielen E., Vanderschueren D. (2011). Testosterone and the male skeleton: A dual mode of action. J. Osteoporos..

[6] Gennari L., Nuti R., Bilezikian J.P. (2004). Aromatase activity and bone homeostasis in men. J. Clin. Endocrinol. Metab..

[7] Vandenput L., Ohlsson C. (2010). Sex steroid metabolism in the regulation of bone health in men. J. Steroid Biochem. Mol. Biol..

[8] Sun L., Peng Y., Sharrow A.C., Iqbal J., Zhang Z., Papachristou D.J., Zaidi S., Zhu L.-L., Yaroslavskiy B.B., Zhou H. (2006). FSH directly regulates bone mass. Cell.

[9] Robinson L.J., Tourkova I., Wang Y., Sharrow A.C., Landau M.S., Yaroslavskiy B.B., Sun L., Zaidi M., Blair H.C. (2010). FSH-receptor isoforms and FSH-dependent gene transcription in human monocytes and osteoclasts. Biochem. Biophys. Res. Commun..

[10] Sun L., Zhang Z., Zhu L.-L., Peng Y., Liu X., Li J., Agrawal M., Robinson L.J., Iqbal J., Blair H.C. (2010). Further evidence for direct pro-resorptive actions of FSH. Biochem. Biophys. Res. Commun..

[11] Wu Y., Torchia J., Yao W., Lane N.E., Lanier L.L., Nakamura M.C., Humphrey M.B. (2007). Bone microenvironment specific roles of ITAM adapter signaling during bone remodeling induced by acute estrogen-deficiency. PLoS ONE.

[12] Iqbal J., Sun L., Kumar T.R., Blair H.C., Zaidi M. (2006). Follicle-stimulating hormone stimulates TNF production from immune cells to enhance osteoblast and osteoclast formation. Proc. Natl. Acad. Sci. USA.

[13] Cannon J.G., Kraj B., Sloan G. (2011). Follicle-stimulating hormone promotes RANK expression on human monocytes. Cytokine.

[14] Meher B.R., Dixit A., Bousfield G.R., Lushington G.H. (2015). Glycosylation Effects on FSH-FSHR Interaction Dynamics: A Case Study of Different FSH Glycoforms by Molecular Dynamics Simulations. PLoS ONE.

[15] Cannon J.G., Cortez-Cooper M., Meaders E., Stallings J., Haddow S., Kraj B., Sloan G., Mulloy A. (2010). Follicle-stimulating hormone, interleukin-1, and bone density in adult women. Am. J. Physiol. Regul. Integr. Comp. Physiol..

[16] Zhu L.-L., Blair H., Cao J., Yuen T., Latif R., Guo L., Tourkova I.L., Li J., Davies T.F., Sun L. (2012). Blocking antibody to the beta-subunit of FSH prevents bone loss by inhibiting bone resorption and stimulating bone synthesis. Proc. Natl. Acad. Sci. USA.

[17] Gallagher C.M., Moonga B.S., Kovach J.S. (2010). Cadmium, follicle-stimulating hormone, and effects on bone in women age 42–60 years, NHANES III. Environ. Res..

[18] Sowers M.R., Greendale G.A., Bondarenko I., Finkelstein J.S., Cauley J.A., Neer R.M., Ettinger B. (2003). Endogenous hormones and bone turnover markers in pre- and perimenopausal women: SWAN. Osteoporos. Int..

[19] Rendina D., Gianfrancesco F., De Filippo G., Merlotti D., Esposito T., Mingione A., Nuti R., Strazzullo P., Mossetti G., Gennari L. (2010). FSHR gene polymorphisms influence bone mineral density and bone turnover in postmenopausal women. Eur. J. Endocrinol..

[20] Kawai H., Furuhashi M., Suganuma N. (2004). Serum follicle-stimulating hormone level is a predictor of bone mineral density in patients with hormone replacement therapy. Arch. Gynecol. Obstet..

[21] Randolph J.F.J., Sowers M., Gold E.B., Mohr B.A., Luborsky J., Santoro N., McConnell D.S., Finkelstein J.S., Korenman S.G., Matthews K.A. (2003). Reproductive hormones in the early menopausal transition: Relationship to ethnicity, body size, and menopausal status. J. Clin. Endocrinol. Metab..

[22] Karim N., MacDonald D., Dolan A.L., Fogelman I., Wierzbicki A.S., Hampson G. (2008). The relationship between gonadotrophins, gonadal hormones and bone mass in men. Clin. Endocrinol..

[23] Ferlin A., Schipilliti M., Vinanzi C., Garolla A., Di Mambro A., Selice R., Lenzi A., Foresta C. (2011). Bone mass in subjects with Klinefelter syndrome: Role of testosterone levels and androgen receptor gene CAG polymorphism. J. Clin. Endocrinol. Metab..

[24] Falchetti A. (2002). Genetics of osteoarticular disorders, Florence, Italy, 22–23 February 2002. Arthritis Res..

[25] Gennari L., Brandi M.L. (2001). Genetics of male osteoporosis. Calcif. Tissue Int..

[26] Van Pottelbergh I., Goemaere S., Zmierczak H., De Bacquer D., Kaufman J.M. (2003). Deficient acquisition of bone during maturation underlies idiopathic osteoporosis in men: Evidence from a three-generation family study. J. Bone Miner. Res..

[27] Smith D.M., Nance W.E., Kang K.W., Christian J.C., Johnston C.C.J. (1973). Genetic factors in determining bone mass. J. Clin. Investig..

[28] Smith E.P., Boyd J., Frank G.R., Takahashi H., Cohen R.M., Specker B., Williams T.C., Lubahn D.B., Korach K.S. (1994). Estrogen resistance caused by a mutation in the estrogen-receptor gene in a man. N. Engl. J. Med..

[29] Gennari L., Masi L., Merlotti D., Picariello L., Falchetti A., Tanini A., Mavilia C., Del Monte F., Gonnelli S., Lucani B. (2004). A polymorphic CYP19 TTTA repeat influences aromatase activity and estrogen levels in elderly men: Effects on bone metabolism. J. Clin. Endocrinol. Metab..

[30] Gennari L., Bilezikian J.P. (2013). Idiopathic osteoporosis in men. Curr. Osteoporos. Rep..

[31] Rosen C.J., Kurland E.S., Vereault D., Adler R.A., Rackoff P.J., Craig W.Y., Witte S., Rogers J., Bilezikian J.P. (1998). Association between serum insulin growth factor-I (IGF-I) and a simple sequence repeat in IGF-I gene: Implications for genetic studies of bone mineral density. J. Clin. Endocrinol. Metab..

[32] Varanasi S.S., Francis R.M., Berger C.E., Papiha S.S., Datta H.K. (1999). Mitochondrial DNA deletion associated oxidative stress and severe male osteoporosis. Osteoporos. Int..

[33] Cosso R., Falchetti A. (2018). Mitochondriopathies and bone health. Trends Biomed. Res..

[34] Trajanoska K., Rivadeneira F. (2019). The genetic architecture of osteoporosis and fracture risk. Bone.

[35] Laine C.M., Joeng K.S., Campeau P.M., Kiviranta R., Tarkkonen K., Grover M., Lu J.T., Pekkinen M., Wessman M., Heino T.J. (2013). WNT1 mutations in early-onset osteoporosis and osteogenesis imperfecta. N. Engl. J. Med..

[36] Lara-Castillo N., Johnson M.L. (2015). LRP receptor family member associated bone disease. Rev. Endocr. Metab. Disord..

[37] Willson T., Nelson S.D., Newbold J., Nelson R.E., LaFleur J. (2015). The clinical epidemiology of male osteoporosis: A review of the recent literature. Clin. Epidemiol..

[38] Gennari L., Bilezikian J.P. (2007). Osteoporosis in men. Endocrinol. Metab. Clin. N. Am..

[39] Frost M., Wraae K., Abrahamsen B., Hoiberg M., Hagen C., Andersen M., Brixen K. (2012). Osteoporosis and vertebral fractures in men aged 60–74 years. Age Ageing.

[40] Blume S.W., Curtis J.R. (2011). Medical costs of osteoporosis in the elderly Medicare population. Osteoporos. Int..

[41] Hernlund E., Svedbom A., Ivergard M., Compston J., Cooper C., Stenmark J., McCloskey E.V., Jonsson B., Kanis J.A. (2013). Osteoporosis in the European Union: Medical management, epidemiology and economic burden. A report prepared in collaboration with the International Osteoporosis Foundation (IOF) and the European Federation of Pharmaceutical Industry Associations (EFPIA). Arch. Osteoporos..

[42] Burge R., Dawson-Hughes B., Solomon D.H., Wong J.B., King A., Tosteson A. (2007). Incidence and economic burden of osteoporosis-related fractures in the United States, 2005–2025. J. Bone Miner. Res..

[43] Johnell O., Kanis J.A. (2006). An estimate of the worldwide prevalence and disability associated with osteoporotic fractures. Osteoporos. Int..

[44] Kim S.H., Meehan J.P., Blumenfeld T., Szabo R.M. (2012). Hip fractures in the United States: 2008 nationwide emergency department sample. Arthritis Care Res..

[45] Diamantopoulos A.P., Rohde G., Johnsrud I., Skoie I.M., Johnsen V., Hochberg M., Haugeberg G. (2012). Incidence rates of fragility hip fracture in middle-aged and elderly men and women in southern Norway. Age Ageing.

[46] Edwards B.J., Bunta A.D., Simonelli C., Bolander M., Fitzpatrick L.A. (2007). Prior fractures are common in patients with subsequent hip fractures. Clin. Orthop. Relat. Res..

[47] Omsland T.K., Holvik K., Meyer H.E., Center J.R., Emaus N., Tell G.S., Schei B., Tverdal A., Gjesdal C.G., Grimnes G. (2012). Hip fractures in Norway 1999–2008: Time trends in total incidence and second hip fracture rates: A NOREPOS study. Eur. J. Epidemiol..

[48] von Friesendorff M., McGuigan F.E., Besjakov J., Akesson K. (2011). Hip fracture in men-survival and subsequent fractures: A cohort study with 22-year follow-up. J. Am. Geriatr. Soc..

[49] Cosman F., de Beur S.J., LeBoff M.S., Lewiecki E.M., Tanner B., Randall S., Lindsay R. (2014). Clinician’s Guide to Prevention and Treatment of Osteoporosis. Osteoporos. Int..

[50] Watts N.B., Adler R.A., Bilezikian J.P., Drake M.T., Eastell R., Orwoll E.S., Finkelstein J.S. (2012). Osteoporosis in men: An Endocrine Society clinical practice guideline. J. Clin. Endocrinol. Metab..

[51] (2011). Screening for osteoporosis: U.S. preventive services task force recommendation statement. Ann. Intern. Med..

[52] Diem S.J., Peters K.W., Gourlay M.L., Schousboe J.T., Taylor B.C., Orwoll E.S., Cauley J.A., Langsetmo L., Crandall C.J., Ensrud K.E. (2017). Screening for Osteoporosis in Older Men: Operating Characteristics of Proposed Strategies for Selecting Men for BMD Testing. J. Gen. Intern. Med..

[53] McCloskey E., Johansson H., Oden A., Kanis J.A. (2012). Fracture risk assessment. Clin. Biochem..

[54] Colon-Emeric C., Pieper C., Lyles K., VanHoutven C., LaFleur J., Adler R. (2017). Primary Osteoporosis Screening in U.S. Male Veterans is Effective in HighRisk Subgroups, but not Overall. J. Bone Miner. Res..

[55] Cauley J.A., Cawthon P.M., Peters K.E., Cummings S.R., Ensrud K.E., Bauer D.C., Taylor B.C., Shikany J.M., Hoffman A.R., Lane N.E. (2016). Risk Factors for Hip Fracture in Older Men: The Osteoporotic Fractures in Men Study (MrOS). J. Bone Miner. Res..

[56] Malmstrom T.K., Morley J.E. (2013). SARC-F: A simple questionnaire to rapidly diagnose sarcopenia. J. Am. Med. Dir. Assoc..

[57] Su Y., Leung J., Kwok T., Cosman F., de Beur S.J., LeBoff M.S., Lewiecki E.M., Tanner B., Randall S., Lindsay R. (2018). The role of previous falls in major osteoporotic fracture prediction in conjunction with FRAX in older Chinese men and women: The Mr. OS and Ms. OS cohort study in Hong Kong. Osteoporos. Int..

[58] Nguyen T. (2018). V Individualized fracture risk assessment: State-of-the-art and room for improvement. Osteoporos. Sarcopenia.

[59] Beaudoin C., Moore L., Gagne M., Bessette L., Ste-Marie L.G., Brown J.P., Jean S. (2019). Performance of predictive tools to identify individuals at risk of non-traumatic fracture: A systematic review, meta-analysis, and meta-regression. Osteoporos. Int..

[60] Leslie W.D., Majumdar S.R., Morin S.N., Lix L.M., Schousboe J.T., Ensrud K.E., Johansson H., McCloskey E.V., Kanis J.A. (2018). Performance of FRAX in clinical practice according to sex and osteoporosis definitions: The Manitoba BMD registry. Osteoporos. Int..

[61] Gourlay M.L., Ritter V.S., Fine J.P., Overman R.A., Schousboe J.T., Cawthon P.M., Orwoll E.S., Nguyen T.V., Lane N.E., Cummings S.R. (2017). Comparison of fracture risk assessment tools in older men without prior hip or spine fracture: The MrOS study. Arch. Osteoporos..

[62] Harvey N.C., Oden A., Orwoll E., Lapidus J., Kwok T., Karlsson M.K., Rosengren B.E., Ribom E., Cooper C., Cawthon P.M. (2018). Measures of Physical Performance and Muscle Strength as Predictors of Fracture Risk Independent of FRAX, Falls, and aBMD: A Meta-Analysis of the Osteoporotic Fractures in Men (MrOS) Study. J. Bone Miner. Res..

[63] Leslie W.D., Johansson H., McCloskey E.V., Harvey N.C., Kanis J.A., Hans D. (2018). Comparison of Methods for Improving Fracture Risk Assessment in Diabetes: The Manitoba BMD Registry. J. Bone Miner. Res..

[64] (2004). Diagnosis of osteoporosis in men, premenopausal women, and children. J. Clin. Densitom..

[65] Center J.R., Bliuc D., Nguyen T.V., Eisman J.A. (2007). Risk of subsequent fracture after low-trauma fracture in men and women. JAMA.

[66] Cummings S.R., Bates D., Black D.M. (2002). Clinical use of bone densitometry: Scientific review. JAMA.

[67] Schousboe J.T., Taylor B.C., Fink H.A., Kane R.L., Cummings S.R., Orwoll E.S., Melton L.J., Bauer D.C., Ensrud K.E. (2007). Cost-effectiveness of bone densitometry followed by treatment of osteoporosis in older men. JAMA.

[68] Gagnon C., Schafer A.L. (2018). Bone Health after Bariatric Surgery. JBMR Plus.

[69] Shuhart C.R., Yeap S.S., Anderson P.A., Jankowski L.G., Lewiecki E.M., Morse L.R., Rosen H.N., Weber D.R., Zemel B.S., Shepherd J.A. (2019). Executive Summary of the 2019 ISCD Position Development Conference on Monitoring Treatment, DXA Cross-calibration and Least Significant Change, Spinal Cord Injury, Peri-prosthetic and Orthopedic Bone Health, Transgender Medicine, and Pediatrics. J. Clin. Densitom..

[70] Khosla S., Amin S., Orwoll E. (2008). Osteoporosis in men. Endocr. Rev..

[71] Kanis J.A., McCloskey E.V., Johansson H., Oden A., Melton L.J., Khaltaev N. (2008). A reference standard for the description of osteoporosis. Bone.

[72] Rossini M., Adami S., Bertoldo F., Diacinti D., Gatti D., Giannini S., Giusti A., Malavolta N., Minisola S., Osella G. (2016). Guidelines for the diagnosis, prevention and management of osteoporosis. Reumatismo.

[73] World Health Organization (2007). Who Scientific Group on the Assessment of Osteoporosis at Primary Health.

[74] Lewis C.E., Ewing S.K., Taylor B.C., Shikany J.M., Fink H.A., Ensrud K.E., Barrett-Connor E., Cummings S.R., Orwoll E. (2007). Predictors of non-spine fracture in elderly men: The MrOS study. J. Bone Miner. Res..

[75] Ensrud K.E., Taylor B.C., Peters K.W., Gourlay M.L., Donaldson M.G., Leslie W.D., Blackwell T.L., Fink H.A., Orwoll E.S., Schousboe J. (2014). Implications of expanding indications for drug treatment to prevent fracture in older men in United States: Cross sectional and longitudinal analysis of prospective cohort study. BMJ.

[76] de Laet C.E.D.H., van der Klift M., Hofman A., Pols H.A.P. (2002). Osteoporosis in men and women: A story about bone mineral density thresholds and hip fracture risk. J. Bone Miner. Res..

[77] Rivadeneira F., Zillikens M.C., De Laet C.E., Hofman A., Uitterlinden A.G., Beck T.J., Pols H.A. (2007). Femoral neck BMD is a strong predictor of hip fracture susceptibility in elderly men and women because it detects cortical bone instability: The Rotterdam Study. J. Bone Miner. Res..

[78] Ebeling P.R. (2008). Clinical practice. Osteoporosis in men. N. Engl. J. Med..

[79] Rao S.S., Budhwar N., Ashfaque A. (2010). Osteoporosis in men. Am. Fam. Phys..

[80] Fink H.A., Litwack-Harrison S., Taylor B.C., Bauer D.C., Orwoll E.S., Lee C.G., Barrett-Connor E., Schousboe J.T., Kado D.M., Garimella P.S. (2016). Clinical utility of routine laboratory testing to identify possible secondary causes in older men with osteoporosis: The Osteoporotic Fractures in Men (MrOS) Study. Osteoporos. Int..

[81] Vescini F., Attanasio R., Balestrieri A., Bandeira F., Bonadonna S., Camozzi V., Cassibba S., Cesareo R., Chiodini I., Francucci C.M. (2016). Italian association of clinical endocrinologists (AME) position statement: Drug therapy of osteoporosis. J. Endocrinol. Investig..

[82] Reginster J.-Y., Abadie E., Delmas P., Rizzoli R., Dere W., der Auwera P., Avouac B., Brandi M.-L., Daifotis A., Diez-Perez A. (2006). Recommendations for an update of the current (2001) regulatory requirements for registration of drugs to be used in the treatment of osteoporosis in postmenopausal women and in men. Osteoporos. Int..

[83] Murad M.H., Drake M.T., Mullan R.J., Mauck K.F., Stuart L.M., Lane M.A., Abu Elnour N.O., Erwin P.J., Hazem A., Puhan M.A. (2012). Clinical review. Comparative effectiveness of drug treatments to prevent fragility fractures: A systematic review and network meta-analysis. J. Clin. Endocrinol. Metab..

[84] Gennari L., Rotatori S., Bianciardi S., Nuti R., Merlotti D. (2016). Treatment needs and current options for postmenopausal osteoporosis. Expert Opin. Pharmacother..

[85] Tosteson A.N.A., Melton L.J., Dawson-Hughes B., Baim S., Favus M.J., Khosla S., Lindsay R.L. (2008). Cost-effective osteoporosis treatment thresholds: The United States perspective. Osteoporos. Int..

[86] Sawka A.M., Papaioannou A., Adachi J.D., Gafni A., Hanley D.A., Thabane L. (2005). Does alendronate reduce the risk of fracture in men? A meta-analysis incorporating prior knowledge of anti-fracture efficacy in women. BMC Musculoskelet. Disord..

[87] Ringe J.D., Farahmand P., Faber H., Dorst A. (2009). Sustained efficacy of risedronate in men with primary and secondary osteoporosis: Results of a 2-year study. Rheumatol. Int..

[88] Smith M.R., Egerdie B., Hernandez Toriz N., Feldman R., Tammela T.L.J., Saad F., Heracek J., Szwedowski M., Ke C., Kupic A. (2009). Denosumab in men receiving androgen-deprivation therapy for prostate cancer. N. Engl. J. Med..

[89] Kaufman J.-M., Orwoll E., Goemaere S., San Martin J., Hossain A., Dalsky G.P., Lindsay R., Mitlak B.H. (2005). Teriparatide effects on vertebral fractures and bone mineral density in men with osteoporosis: Treatment and discontinuation of therapy. Osteoporos. Int..

[90] Orwoll E., Ettinger M., Weiss S., Miller P., Kendler D., Graham J., Adami S., Weber K., Lorenc R., Pietschmann P. (2000). Alendronate for the treatment of osteoporosis in men. N. Engl. J. Med..

[91] Gonnelli S., Cepollaro C., Montagnani A., Bruni D., Caffarelli C., Breschi M., Gennari L., Gennari C., Nuti R. (2003). Alendronate treatment in men with primary osteoporosis: A three-year longitudinal study. Calcif. Tissue Int..

[92] Miller P.D., Schnitzer T., Emkey R., Orwoll E., Rosen C., Ettinger M., Vandormael K., Daifotis A. (2004). Weekly oral alendronic Acid in male osteoporosis. Clin. Drug Investig..

[93] Boonen S., Lorenc R.S., Wenderoth D., Stoner K.J., Eusebio R., Orwoll E.S. (2012). Evidence for safety and efficacy of risedronate in men with osteoporosis over 4 years of treatment: Results from the 2-year, open-label, extension study of a 2-year, randomized, double-blind, placebo-controlled study. Bone.

[94] Boonen S., Orwoll E.S., Wenderoth D., Stoner K.J., Eusebio R., Delmas P.D. (2009). Once-weekly risedronate in men with osteoporosis: Results of a 2-year, placebo-controlled, double-blind, multicenter study. J. Bone Miner. Res..

[95] Orwoll E.S., Binkley N.C., Lewiecki E.M., Gruntmanis U., Fries M.A., Dasic G. (2010). Efficacy and safety of monthly ibandronate in men with low bone density. Bone.

[96] Boonen S., Reginster J.-Y., Kaufman J.-M., Lippuner K., Zanchetta J., Langdahl B., Rizzoli R., Lipschitz S., Dimai H.P., Witvrouw R. (2012). Fracture risk and zoledronic acid therapy in men with osteoporosis. N. Engl. J. Med..

[97] Boonen S., Orwoll E., Magaziner J., Colon-Emeric C.S., Adachi J.D., Bucci-Rechtweg C., Haentjens P., Kaufman J.-M., Rizzoli R., Vanderschueren D. (2011). Once-yearly zoledronic acid in older men compared with women with recent hip fracture. J. Am. Geriatr. Soc..

[98] Smith M.R., Saad F., Coleman R., Shore N., Fizazi K., Tombal B., Miller K., Sieber P., Karsh L., Damiao R. (2012). Denosumab and bone-metastasis-free survival in men with castration-resistant prostate cancer: Results of a phase 3, randomised, placebo-controlled trial. Lancet.

[99] Orwoll E., Teglbjaerg C.S., Langdahl B.L., Chapurlat R., Czerwinski E., Kendler D.L., Reginster J.-Y., Kivitz A., Lewiecki E.M., Miller P.D. (2012). A randomized, placebo-controlled study of the effects of denosumab for the treatment of men with low bone mineral density. J. Clin. Endocrinol. Metab..

[100] Cummings S.R., Ferrari S., Eastell R., Gilchrist N., Jensen J.-E.B., McClung M., Roux C., Torring O., Valter I., Wang A.T. (2018). Vertebral Fractures After Discontinuation of Denosumab: A Post Hoc Analysis of the Randomized Placebo-Controlled FREEDOM Trial and Its Extension. J. Bone Miner. Res..

[101] Kurland E.S., Cosman F., McMahon D.J., Rosen C.J., Lindsay R., Bilezikian J.P. (2000). Parathyroid hormone as a therapy for idiopathic osteoporosis in men: Effects on bone mineral density and bone markers. J. Clin. Endocrinol. Metab..

[102] Orwoll E.S., Scheele W.H., Paul S., Adami S., Syversen U., Diez-Perez A., Kaufman J.M., Clancy A.D., Gaich G.A. (2003). The effect of teriparatide [human parathyroid hormone (1–34)] therapy on bone density in men with osteoporosis. J. Bone Miner. Res..

[103] Slovik D.M., Rosenthal D.I., Doppelt S.H., Potts J.T.J., Daly M.A., Campbell J.A., Neer R.M. (1986). Restoration of spinal bone in osteoporotic men by treatment with human parathyroid hormone (1–34) and 1,25-dihydroxyvitamin D. J. Bone Miner. Res..

[104] Saag K.G., Shane E., Boonen S., Marin F., Donley D.W., Taylor K.A., Dalsky G.P., Marcus R. (2007). Teriparatide or alendronate in glucocorticoid-induced osteoporosis. N. Engl. J. Med..

[105] Saag K.G., Zanchetta J.R., Devogelaer J.-P., Adler R.A., Eastell R., See K., Krege J.H., Krohn K., Warner M.R. (2009). Effects of teriparatide versus alendronate for treating glucocorticoid-induced osteoporosis: Thirty-six-month results of a randomized, double-blind, controlled trial. Arthritis Rheum..

[106] Schwartz A.V., Pavo I., Alam J., Disch D.P., Schuster D., Harris J.M., Krege J.H. (2016). Teriparatide in patients with osteoporosis and type 2 diabetes. Bone.

[107] Tracz M.J., Sideras K., Bolona E.R., Haddad R.M., Kennedy C.C., Uraga M.V., Caples S.M., Erwin P.J., Montori V.M. (2006). Testosterone use in men and its effects on bone health. A systematic review and meta-analysis of randomized placebo-controlled trials. J. Clin. Endocrinol. Metab..

[108] Anderson F.H., Francis R.M., Peaston R.T., Wastell H.J. (1997). Androgen supplementation in eugonadal men with osteoporosis: Effects of six months’ treatment on markers of bone formation and resorption. J. Bone Miner. Res..

[109] Basaria S., Coviello A.D., Travison T.G., Storer T.W., Farwell W.R., Jette A.M., Eder R., Tennstedt S., Ulloor J., Zhang A. (2010). Adverse events associated with testosterone administration. N. Engl. J. Med..

[110] Walker R.F., Zakai N.A., MacLehose R.F., Cowan L.T., Adam T.J., Alonso A., Lutsey P.L. (2019). Association of Testosterone Therapy With Risk of Venous Thromboembolism Among Men With and Without Hypogonadism. JAMA Intern. Med..

[111] Uebelhart B., Herrmann F., Pavo I., Draper M.W., Rizzoli R. (2004). Raloxifene treatment is associated with increased serum estradiol and decreased bone remodeling in healthy middle-aged men with low sex hormone levels. J. Bone Miner. Res..

[112] Smith M.R., Morton R.A., Barnette K.G., Sieber P.R., Malkowicz S.B., Rodriguez D., Hancock M.L., Steiner M.S. (2013). Toremifene to reduce fracture risk in men receiving androgen deprivation therapy for prostate cancer. J. Urol..

[113] Gennari L., Merlotti D., Falchetti A., Eller Vainicher C., Cosso R., Chiodini I. (2020). Emerging therapeutic targets for osteoporosis. Expert Opin. Ther. Targets.

[114] Costa A.G., Cusano N.E., Silva B.C., Cremers S., Bilezikian J.P. (2011). Cathepsin K: Its skeletal actions and role as a therapeutic target in osteoporosis. Nat. Rev. Rheumatol..

[115] Holliday L.S. (2017). Vacuolar H(+)-ATPases (V-ATPases) as therapeutic targets: A brief review and recent developments. Biotarget.

[116] Liu X., Qu X., Nie T., Zhai Z., Li H., Ouyang Z., Qin A., Zhang S., Zhang S., Fan Q. (2017). The Beneficial Effects of Bisphosphonate-enoxacin on Cortical Bone Mass and Strength in Ovariectomized Rats. Front. Pharmacol..

[117] Soriano P., Montgomery C., Geske R., Bradley A. (1991). Targeted disruption of the c-src proto-oncogene leads to osteopetrosis in mice. Cell.

[118] Marzia M., Sims N.A., Voit S., Migliaccio S., Taranta A., Bernardini S., Faraggiana T., Yoneda T., Mundy G.R., Boyce B.F. (2000). Decreased c-Src expression enhances osteoblast differentiation and bone formation. J. Cell Biol..

[119] Hannon R.A., Clack G., Rimmer M., Swaisland A., Lockton J.A., Finkelman R.D., Eastell R. (2010). Effects of the Src kinase inhibitor saracatinib (AZD0530) on bone turnover in healthy men: A randomized, double-blind, placebo-controlled, multiple-ascending-dose phase I trial. J. Bone Miner. Res..

[120] Lewiecki E.M., Blicharski T., Goemaere S., Lippuner K., Meisner P.D., Miller P.D., Miyauchi A., Maddox J., Chen L., Horlait S. (2018). A Phase III Randomized Placebo-Controlled Trial to Evaluate Efficacy and Safety of Romosozumab in Men With Osteoporosis. J. Clin. Endocrinol. Metab..

[121] Glantschnig H., Scott K., Hampton R., Wei N., McCracken P., Nantermet P., Zhao J.Z., Vitelli S., Huang L., Haytko P. (2011). A rate-limiting role for Dickkopf-1 in bone formation and the remediation of bone loss in mouse and primate models of postmenopausal osteoporosis by an experimental therapeutic antibody. J. Pharmacol. Exp. Ther..

[122] Florio M., Gunasekaran K., Stolina M., Li X., Liu L., Tipton B., Salimi-Moosavi H., Asuncion F.J., Li C., Sun B. (2016). A bispecific antibody targeting sclerostin and DKK-1 promotes bone mass accrual and fracture repair. Nat. Commun..

[123] Zainabadi K., Liu C.J., Caldwell A.L.M., Guarente L. (2017). SIRT1 is a positive regulator of in vivo bone mass and a therapeutic target for osteoporosis. PLoS ONE.

[124] Ornstrup M.J., Harslof T., Kjaer T.N., Langdahl B.L., Pedersen S.B. (2014). Resveratrol increases bone mineral density and bone alkaline phosphatase in obese men: A randomized placebo-controlled trial. J. Clin. Endocrinol. Metab..

[125] Joshua J., Schwaerzer G.K., Kalyanaraman H., Cory E., Sah R.L., Li M., Vaida F., Boss G.R., Pilz R.B. (2014). Soluble guanylate cyclase as a novel treatment target for osteoporosis. Endocrinology.

[126] Napoli N., Chandran M., Pierroz D.D., Abrahamsen B., Schwartz A.V., Ferrari S.L. (2017). Mechanisms of diabetes mellitus-induced bone fragility. Nat. Rev. Endocrinol..

[127] Kalyanaraman H., Schwaerzer G., Ramdani G., Castillo F., Scott B.T., Dillmann W., Sah R.L., Casteel D.E., Pilz R.B. (2018). Protein Kinase G Activation Reverses Oxidative Stress and Restores Osteoblast Function and Bone Formation in Male Mice With Type 1 Diabetes. Diabetes.

[128] Anastasilakis A.D., Polyzos S.A., Makras P., Gkiomisi A., Savvides M., Papatheodorou A., Terpos E. (2013). Circulating activin-A is elevated in postmenopausal women with low bone mass: The three-month effect of zoledronic acid treatment. Osteoporos. Int..

[129] Lotinun S., Pearsall R.S., Horne W.C., Baron R. (2012). Activin receptor signaling: A potential therapeutic target for osteoporosis. Curr. Mol. Pharmacol..

[130] Lotinun S., Pearsall R.S., Davies M.V., Marvell T.H., Monnell T.E., Ucran J., Fajardo R.J., Kumar R., Underwood K.W., Seehra J. (2010). A soluble activin receptor Type IIA fusion protein (ACE-011) increases bone mass via a dual anabolic-antiresorptive effect in Cynomolgus monkeys. Bone.

[131] Ruckle J., Jacobs M., Kramer W., Pearsall A.E., Kumar R., Underwood K.W., Seehra J., Yang Y., Condon C.H., Sherman M.L. (2009). Single-dose, randomized, double-blind, placebo-controlled study of ACE-011 (ActRIIA-IgG1) in postmenopausal women. J. Bone Miner. Res..

[132] Hayashi M., Nakashima T., Taniguchi M., Kodama T., Kumanogoh A., Takayanagi H. (2012). Osteoprotection by semaphorin 3A. Nature.

[133] Zhang Y., Wei L., Miron R.J., Shi B., Bian Z. (2015). Anabolic bone formation via a site-specific bone-targeting delivery system by interfering with semaphorin 4D expression. J. Bone Miner. Res..

[134] Grassi F., Tyagi A.M., Calvert J.W., Gambari L., Walker L.D., Yu M., Robinson J., Li J.-Y., Lisignoli G., Vaccaro C. (2016). Hydrogen Sulfide Is a Novel Regulator of Bone Formation Implicated in the Bone Loss Induced by Estrogen Deficiency. J. Bone Miner. Res..

[135] Rapposelli S., Gambari L., Digiacomo M., Citi V., Lisignoli G., Manferdini C., Calderone V., Grassi F. (2017). A Novel H2S-releasing Amino-Bisphosphonate which combines bone anti-catabolic and anabolic functions. Sci. Rep..

[136] Refaey M.E., McGee-Lawrence M.E., Fulzele S., Kennedy E.J., Bollag W.B., Elsalanty M., Zhong Q., Ding K.-H., Bendzunas N.G., Shi X.-M. (2017). Kynurenine, a Tryptophan Metabolite That Accumulates With Age, Induces Bone Loss. J. Bone Miner. Res..

[137] Bozec A., Zaiss M.M., Kagwiria R., Voll R., Rauh M., Chen Z., Mueller-Schmucker S., Kroczek R.A., Heinzerling L., Moser M. (2014). T cell costimulation molecules CD80/86 inhibit osteoclast differentiation by inducing the IDO/tryptophan pathway. Sci. Transl. Med..

[138] Vidal C., Li W., Santner-Nanan B., Lim C.K., Guillemin G.J., Ball H.J., Hunt N.H., Nanan R., Duque G. (2015). The kynurenine pathway of tryptophan degradation is activated during osteoblastogenesis. Stem Cells.

[139] Hu J., Zheng W., Zhao D., Sun L., Zhou B., Liu J., Wang O., Jiang Y., Xia W., Xing X. (2021). Health-related quality of life in men with osteoporosis: A systematic review and meta-analysis. Endocrine.

